# Extracellular Matrix in Human Disease and Therapy: From Pathogenic Remodeling to Biomaterial Platforms and Precision Diagnostics

**DOI:** 10.3390/biomedicines14010247

**Published:** 2026-01-21

**Authors:** Jun-Hyeog Jang

**Affiliations:** Department of Biochemistry, Inha University School of Medicine, Incheon 22212, Republic of Korea; juhjang@inha.ac.kr; Fax: +82-32-882-1877

**Keywords:** extracellular matrix, mechanotransduction, fibrosis, tumor microenvironment, decellularized ECM, hydrogels, biomaterials, biomarkers, imaging, precision medicine

## Abstract

The extracellular matrix (ECM) is a dynamic, tissue-specific network that integrates biochemical and mechanical cues to regulate cell behavior and organ homeostasis. Increasing evidence indicates that dysregulated ECM remodeling is an upstream driver of chronic human diseases rather than a passive consequence of injury. This review summarizes principles of ECM organization, mechanotransduction, and pathological remodeling and highlights translational opportunities for ECM-targeted therapies, biomaterial platforms, and precision diagnostics. We conducted a narrative synthesis of foundational and recent literature covering ECM composition and turnover, stiffness-dependent signaling, and disease-associated remodeling across fibrosis/cardiovascular disease, cancer, and metabolic disorders, together with advances in ECM-based biomaterials, drug delivery, and ECMderived biomarkers and imaging. Across organs, a self-reinforcing cycle of altered matrix composition, excessive crosslinking, and stiffness-dependent mechanotransduction (including integrin–FAK and YAP/TAZ pathways) sustains fibroinflammation, myofibroblast persistence, and progressive tissue dysfunction. In tumors, aligned and crosslinked ECM promotes invasion, immune evasion, and therapy resistance while also shaping perfusion and drug penetration. Translational strategies increasingly focus on modulating ECM synthesis and crosslinking, normalizing rather than ablating matrix architecture, and targeting ECM–cell signaling axes in combination with anti-fibrotic, cytotoxic, or immunotherapeutic regimens. ECM biology provides a unifying framework linking pathogenesis, therapy, and precision diagnostics across chronic diseases. Clinical translation will benefit from standardized quantitative measures of matrix remodeling, mechanism-based biomarkers of ECM turnover, and integrative imaging–omics approaches for patient stratification and treatment monitoring.

## 1. Introduction

The extracellular matrix (ECM) is a three-dimensional macromolecular network composed of collagens, elastin, proteoglycans, and glycoproteins that provides structural integrity and instructive biochemical and biophysical cues to resident cells. Far from being an inert scaffold, the ECM is a dynamic and finely regulated component of the tissue microenvironment that controls cell adhesion, migration, proliferation, differentiation, and survival through a combination of biochemical signaling and mechanotransduction pathways [1,2,3]. In this review, we frame ECM remodeling as a shared, quantifiable mechanobiological axis across major chronic diseases and highlight ECM normalization as a unifying translational principle that connects mechanism-based therapeutics, ECM-informed biomaterials, and precision diagnostics.

In healthy tissues, ECM synthesis, crosslinking, and degradation are tightly balanced by matrix-producing cells and matrix-remodeling enzymes, maintaining tissue-specific architecture and mechanical properties [2,4]. In chronic disease, this equilibrium is disrupted, leading to pathological ECM stiffening, fibrosis, calcification, or excessive degradation that actively drives disease progression rather than merely reflecting downstream damage [4,5,6,7,8,9,10,11].

Recent comprehensive reviews highlight ECM dysregulation as a central hub linking aging, chronic inflammation, fibrosis, calcification, and tumorigenesis [2,4,12]. For example, sustained fibroblast activation and maladaptive matrix deposition support progressive organ fibrosis [5,12], while matrix stiffening and altered topography promote myofibroblast persistence, endothelial dysfunction, and immune cell skewing [4,6,10,11,13,14,15].

Concurrently, the ECM is emerging as an attractive therapeutic and diagnostic axis in translational biomedicine. ECM-derived cues can be harnessed to engineer tissue-specific biomaterials and decellularized scaffolds for regeneration [16,17,18,19,20], while ECM fragments and neo-epitopes serve as minimally invasive biomarkers reflecting dynamic tissue re-modeling [4,21]. Advances in ECM-mimetic hydrogels, nanomaterial-based matrices, and matrisome-informed diagnostics align closely with the aims of translational biomedicine in developing novel biopharmaceutical products, targeted therapies, and mechanistically grounded biomarkers [8,22,23,24,25,26,27,28].

**Scheme 1:**
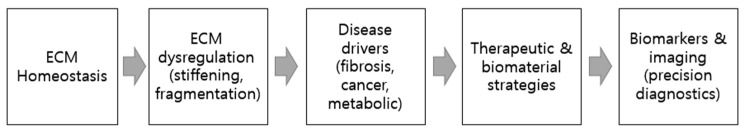
Graphical overview of the review: ECM remodeling as a cross-disease mechanobiological axis.

This review provides a disease-focused overview of ECM biology and its translational exploitation: (i) fundamental principles of ECM organization and remodeling in health and disease; (ii) ECM as an active driver in key human pathologies; (iii) ECM-targeted therapeutic strategies; (iv) ECM-based biomaterials and decellularized scaffolds; and (v) ECM-derived biomarkers, imaging, and integrative diagnostics with relevance to personalized medicine [1,2,8,21,23,26,27]. Compared with prior reviews that focus on single disease settings or individual translational modalities, we frame ECM remodeling as a shared, quantifiable mechanobiological axis across chronic diseases and highlight “ECM normalization” as a unifying translational principle linking therapeutics, biomaterials, and precision diagnostics (Scheme 1) [2,4,11,22,29,30,31,32,33].

## 2. ECM Organization and Remodeling in Health and Disease

### 2.1. Structural and Compositional Principles

**Figure 1:**
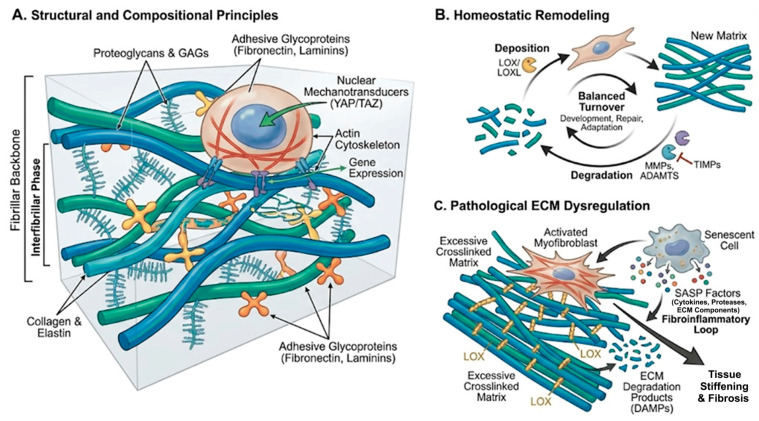
Structural principles and dynamic remodeling of the extracellular matrix (ECM) in homeostasis and disease. (**A**) Structural and compositional organization of the ECM, highlighting collagen/elastin networks, proteoglycans and glycosaminoglycans (GAGs), and adhesive glycoproteins (e.g., fibronectin and laminins) that integrate with the actin cytoskeleton to regulate mechanotransduction (YAP/TAZ) and downstream gene expression. (**B**) Homeostatic ECM remodeling maintained by balanced turnover through coordinated matrix deposition and crosslinking (e.g., LOX/LOXL) and proteolytic degradation (e.g., MMPs and ADAMTS), regulated by endogenous inhibitors (TIMPs) during development, repair, and adaptation. (**C**) Pathological ECM dysregulation characterized by excessive crosslinking and stiffening, activated myofibroblasts, and senescent cell–derived SASP factors that amplify fibroinflammatory loops; ECM degradation products (DAMPs) further propagate inflammation, culminating in tissue stiffening and fibrosis.

The ECM consists of a fibrillar backbone (primarily fibrillar collagens and elastin), an interfibrillar phase of proteoglycans and glycosaminoglycans, and adhesive glycoproteins such as fibronectin and laminins (Figure 1A) [1,2]. These components assemble into tissue-specific architectures that define porosity, viscoelasticity, and anisotropy, thereby shaping cell–matrix interactions. Cells sense the ECM via integrins and other receptors, forming focal adhesions that couple to the actin cytoskeleton and to nuclear mechanotransducers such as YAP/TAZ. This mechanochemical coupling enables cells to translate matrix stiffness, topography, and strain into gene-expression programs and lineage decisions [2,6,13,34,35].

### 2.2. Homeostatic Remodeling

Under physiological conditions, the ECM is continuously remodeled by matrix metalloproteinases (MMPs), ADAMTS proteases, and crosslinking enzymes such as lysyl oxidases (LOX/LOXL), balanced by tissue inhibitors of metalloproteinases (TIMPs) (Figure 1B) [2,7,8,9,12,36]. Controlled deposition and degradation facilitate development, wound repair, and adaptation to mechanical load.

### 2.3. Pathological ECM Dysregulation

Chronic injury, metabolic stress, and aging induce persistent activation of matrix-producing cells (e.g., fibroblasts, myofibroblasts, vascular smooth muscle cells), skewing the balance towards excessive crosslinked matrix deposition, altered composition, and accumulation of bioactive matrix fragments (Figure 1C) [4,5,12]. Senescent cells with senescence-associated secretory phenotypes (SASP) secrete pro-fibrotic cytokines, proteases, and ECM components that perpetuate a fibroinflammatory loop [4]. Systemic accumulation of ECM degradation products further amplifies inflammation and can act as damage-associated molecular patterns (DAMPs) [4]. Nyström and colleagues emphasized that aging, ECM remodeling, and fibrosis are tightly intertwined, with age-related ECM stiffening predisposing tissues to chronic fibrotic remodeling and impaired resolution [13].

Beyond fibrosis, aging itself is accompanied by progressive ECM “hardening” and compositional drift, driven by non-enzymatic glycation/advanced glycation end-product (AGE) crosslinks, elastin fragmentation, impaired proteostasis, and a pro-inflammatory senescence-associated secretory phenotype that remodels the matrisome and raises basal mechanotransduction tone. Emerging interventional studies suggest that targeting the aging ECM may be feasible: senolytic therapy has shown first-in-human signals in idiopathic pulmonary fibrosis, consistent with reducing senescence-linked profibrotic ECM remodeling [37], and AGE crosslink–modifying approaches (e.g., alagebrium/ALT-711) have been evaluated clinically to improve vascular and myocardial compliance [38].

A self-sustaining cycle of ECM stiffening and mechanotransduction drives this pathological state, where increased stiffness promotes further fibroblast activation and matrix deposition. These concepts are reflected across organ systems, including lung, liver, kidney, cardiovascular system, and skin [3,4,5,6,7,8,9,10,11,12,13,14,15].

## 3. ECM as an Active Driver in Major Human Diseases

ECM remodeling is an active driver of pathology by reshaping tissue mechanics and cell signaling across organs. Table 1 summarizes representative ECM alterations and their translational implications across major disease contexts. The following subsections outline key disease settings in which these mechanisms are most evident.

### 3.1. Fibrotic and Cardiovascular Diseases

**Figure 2:**
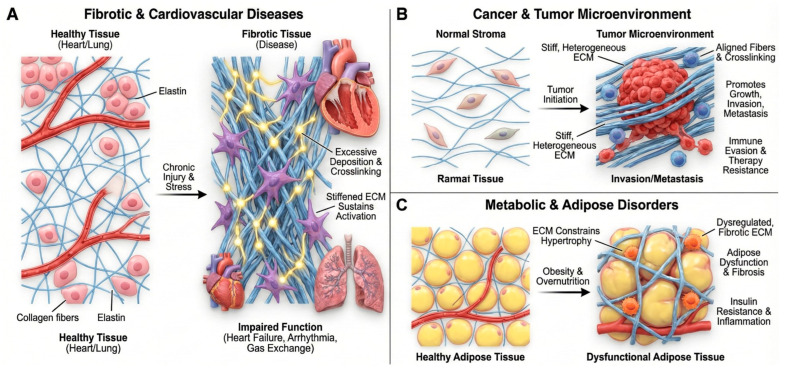
ECM as an active driver in major human diseases. (**A**) Fibrotic and cardiovascular diseases: chronic injury and mechanical stress promote excessive extracellular matrix (ECM) deposition and crosslinking, leading to ECM stiffening, sustained fibroblast/myofibroblast activation, and impaired organ function (e.g., heart failure, arrhythmia, and compromised gas exchange in lung). (**B**) Cancer and the tumor microenvironment: remodeling toward a stiff, heterogeneous, aligned, and highly crosslinked ECM facilitates tumor initiation and supports invasion/metastasis, while contributing to immune evasion and therapy resistance. (**C**) Metabolic and adipose disorders: obesity/overnutrition and ECM remodeling constrain adipocyte hypertrophy and drive a dysregulated, fibrotic ECM, resulting in adipose tissue dysfunction, inflammation, and insulin resistance.

Fibrosis is characterized by excessive ECM deposition, altered crosslinking, and loss of normal matrix architecture, resulting in increased tissue stiffness and impaired function (Figure 2A). Mechanistic studies support a model in which stiffened ECM sustains myofibroblast activation and perpetuates fibrogenic signaling, thereby acting as a driver of progressive fibrosis [5,12]. In cardiac disease, pressure overload, ischemia, and metabolic stress trigger fibroblast activation and collagen-rich scar formation. ECM remodeling alters ventricular compliance, electrical conduction, and mechanosensing, contributing to heart failure and arrhythmia [14]. Reviews of cardiac fibrosis highlight ECM as both a mediator and a potential therapeutic target, including strategies to modulate collagen crosslinking, TGF-*β* signaling, and matricellular proteins [14]. Similarly, in pulmonary fibrosis, aberrant ECM deposition and stiffened foci create a profibrotic microenvironment that amplifies fibroblast activation, distorts alveolar architecture, and compromises gas exchange [5,6,7,8,9,10,11,12,15].

### 3.2. Cancer and the Tumor Microenvironment

The tumor-associated ECM is structurally and compositionally distinct from normal stroma (Figure 2B). Increased collagen density, aligned fibers, enhanced crosslinking, and deposition of matricellular proteins collectively create a stiff, heterogeneous environment that promotes tumor growth, invasion, and immune evasion [29,30,31,32]. Sleeboom et al. proposed the ECM as a “hallmark” of cancer and metastasis, integrating mechanical, biochemical, and topological cues that govern cancer-cell plasticity, migration routes, and access to vasculature [29]. ECM remodeling enzymes and crosslinking pathways (e.g., LOX/LOXL, MMPs) shape metastatic niches and influence response to chemotherapy and immunotherapy [29,30,31]. The interplay between ECM and therapy is bidirectional: cytotoxic and targeted therapies remodel ECM composition and stiffness, while pre-existing ECM states regulate drug penetration, mechanosensitive signaling (e.g., FAK, YAP/TAZ), and immune-cell infiltration [30,32]. Biophysical analyses highlight that ECM stiffness and architecture modulate T cell and NK cell migration, checkpoint expression, and susceptibility to immunotherapies [6,7,8,9,32,34,36,39,44].

### 3.3. Metabolic, Endocrine, and Adipose Tissue Disorders

Adipose tissue expansion in obesity is accompanied by ECM remodeling that constrains adipocyte hypertrophy, impairs vascularization, and sustains low-grade inflammation (Figure 2C). A recent review on adipose tissue development and ECM emphasizes that dysregulated matrix deposition and crosslinking are key determinants of adipose dysfunction, insulin resistance, and fibrosis in obesity and metabolic syndrome [7,8,9,10,11,40]. These findings position ECM as an upstream regulator of endocrine and metabolic homeostasis, linking biomechanical and immunometabolic pathways relevant to cardiovascular and hepatic complications of obesity.

Across fibrosis, cancer, and metabolic disease, recurrent ECM programs—stiffening/crosslinking, aligned fibrillar architecture, and protease-driven remodeling—converge on shared mechanotransduction axes (integrin–FAK and YAP/TAZ) that couple matrix state to inflammatory and pro-survival signaling. However, the strength of evidence and the translational levers differ by context: in fibrosis, feed-forward stiffening–myofibroblast loops are strongly supported by mechanistic and in vivo studies, whereas in cancer, “normalization” strategies must balance improved delivery against context-dependent risks of invasion, underscoring the need for quantitative ECM biomarkers and imaging readouts to guide clinical deployment [3,4,5,6,12,29,30,31,32,34,39,44].

## 4. The ECM as a Therapeutic Target

Given its central role in disease initiation and progression, the ECM is increasingly targeted by pharmacological, biological, and device-based interventions [4,22].

### 4.1. Modulating ECM Synthesis and Crosslinking

**Figure 3:**
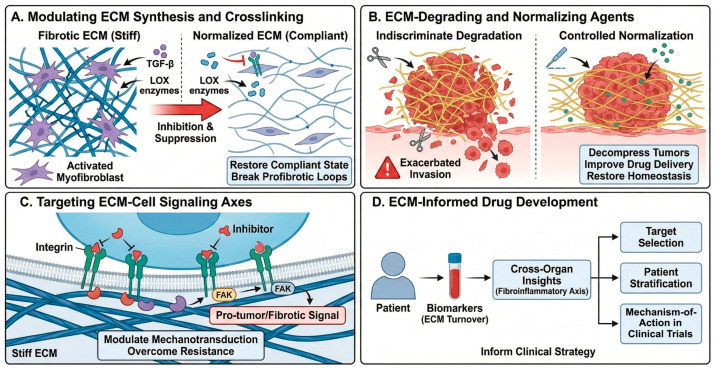
The ECM as a therapeutic target. (**A**) Modulating ECM synthesis and crosslinking to shift a fibrotic, stiff ECM toward a normalized, compliant state by suppressing profibrotic signaling (e.g., TGF-*β*) and enzymatic crosslinking (e.g., LOX), thereby restoring tissue compliance and breaking profibrotic feedback loops. (**B**) ECM-degrading and normalizing strategies: indiscriminate ECM degradation may exacerbate invasion, whereas controlled normalization can decompress tumors, improve drug delivery, and restore tissue homeostasis. (**C**) Targeting ECM–cell signaling axes to modulate mechanotransduction (e.g., integrin–FAK signaling) and overcome pro-tumor/profibrotic signaling and therapy resistance. (**D**) ECM-informed drug development framework in which biomarkers of ECM turnover and cross-organ fibroinflammatory insights guide target selection, patient stratification, and mechanism-of-action assessment in clinical trials.

Anti-fibrotic strategies include inhibition of TGF-*β* signaling, suppression of myofibroblast activation, and targeting of crosslinking enzymes such as LOX/LOXL to reduce excessive collagen crosslinking and stiffness (Figure 3A) [4,12]. These approaches aim to restore more compliant matrix states and break profibrotic feedback loops. Boaru et al. highlighted potential therapeutic avenues targeting senescence-associated ECM remodeling and calcification, such as senolytics, anti-inflammatory agents, and regulators of mineralization [4,6,7,8,9,15].

### 4.2. ECM-Degrading and ECM-Normalizing Agents

ECM-degrading agents (e.g., specific hyaluronidases, collagenases) can decompress solid tumors, improve perfusion, and enhance delivery of chemotherapy and immunotherapy when applied in a controlled manner (Figure 3B) [29,30]. However, indiscriminate degradation may exacerbate invasion or metastasis; thus, “ECM normalization” rather than “ECM ablation” is now favored, aiming to restore a more homeostatic matrix architec-ture [29,30,31]. Importantly, while stromal decompression and controlled ECM normalization show strong preclinical rationale, clinical translation remains context-dependent, and indiscriminate ECM degradation can exacerbate invasion or compromise essential repair processes; therefore, dose, timing, and patient selection should be guided by quantitative ECM readouts (turnover biomarkers and ECM-targeted imaging).

### 4.3. Targeting ECM–Cell Signaling Axes

Inhibitors of integrins and focal adhesion kinase (FAK) are under investigation as mechanotransduction modulators in cancer and fibrotic disease (Figure 3C). A recent study by Betriu et al. demonstrated that increased stiffness in a 3D peptide scaffold model of pancreatic ductal adenocarcinoma (PDAC) downregulated FAK expression and altered mechanosignaling, underscoring matrix–FAK feedback in tumor biology [45]. Combination regimens that pair ECM-modulating agents with immune checkpoint blockade, anti-angiogenic therapies, or targeted kinase inhibitors are being explored to overcome ECM-mediated resistance [3,6,22,30,31,32,39].

### 4.4. ECM-Informed Drug Development

Karsdal et al. [21] synthesized cross-organ insights into ECM-associated diagnostics and therapeutics, proposing a “fibroinflammatory axis” as a common denominator across >50 chronic diseases (Figure 3D) [22]. Their framework emphasizes that understanding ECM turnover and cross-tissue signatures can inform target selection, patient stratification, and mechanism-of-action biomarkers in early-phase clinical trials.

### 4.5. Clinical Translation and Emerging Trials

Although many ECM-targeting strategies remain preclinical, several have advanced to clinical evaluation. In idiopathic pulmonary fibrosis, the LOXL2-blocking antibody simtuzumab was tested in a randomized phase 2 trial, illustrating both the feasibility of targeting matrix crosslinking and the importance of patient stratification and pharmacodynamic biomarkers for ECM engagement [46]. In metastatic pancreatic ductal adenocarcinoma, enzymatic hyaluronan depletion with pegvorhyaluronidase alfa (PEGPH20) was evaluated in the phase 3 HALO 109-301 trial to improve perfusion and chemotherapy delivery—an archetype of controlled matrix decompression/normalization rather than indiscriminate ECM ablation [47]. Mechanotransduction inhibitors have also entered clinical testing; for example, the focal adhesion kinase (FAK) inhibitor defactinib has been assessed in randomized phase 2 studies as maintenance therapy in malignant pleural mesothelioma [48]. Table 2 summarizes representative clinical-stage ECM-modulating approaches and their translational rationale.

## 5. ECM-Based Biomaterials and Decellularized Scaffolds

Biomaterial science has increasingly turned to the ECM as both a blueprint and a raw material for developing regenerative scaffolds and advanced therapeutic products [16,17,18,19,20,23,33,43].

### 5.1. ECM-Derived Biomaterials

**Figure 4:**
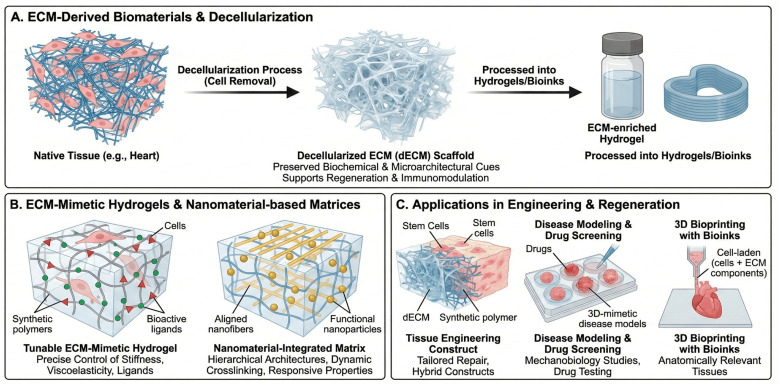
ECM-based biomaterials and decellularized scaffolds. (**A**) Generation of decellularized extracellular matrix (dECM) scaffolds from native tissues via decellularization (cell removal), preserving key biochemical and microarchitectural cues that support regeneration and immunomodulation; dECM can be further processed into ECM-enriched hydrogels and bioinks. (**B**) Engineering ECMmimetic matrices using tunable synthetic polymer hydrogels functionalized with bioactive ligands, and nanomaterial-integrated matrices incorporating aligned nanofibers or functional nanoparticles to control stiffness, viscoelasticity, crosslinking, and dynamic/response properties. (**C**) Applications in tissue engineering and regeneration, including hybrid constructs combining dECM with synthetic polymers for tailored repair, 3D-mimetic disease models for mechanobiology studies and drug screening, and 3D bioprinting with bioinks to fabricate anatomically relevant, cell-laden tissues.

ECM-derived materials, including decellularized tissues and ECM-enriched hydrogels, preserve many native biochemical motifs and microarchitectures that support cell adhesion, migration, and lineage specification (Figure 4A) [16,17,18]. Hussey et al. outlined how ECM-based materials provide instructive cues for regenerative medicine, highlighting their capacity to promote constructive remodeling and immunomodulation [17]. Noro and Vilaça-Faria recently reviewed ECM-derived materials for tissue engineering and regenerative medicine, describing approaches to decellularization, recellularization, and engineering of ECM-based scaffolds tailored to specific tissues [16]. Long et al. emphasized that decellularized ECM (dECM) scaffolds can bridge regeneration and inflammation, depending on how they are prepared and integrated into host tissues [18]. Jin and co-workers further discussed acellular ECM scaffolds as versatile platforms for organ repair, including heart, liver, and musculoskeletal tissues, providing structural templates and bioactive signals in preclinical models [8,19,26,27].

### 5.2. ECM Scaffolds in Tissue Engineering and Regeneration

An in-depth review by Mangani et al. systematically addressed the design and applications of ECM scaffolds in tissue engineering and regeneration, including strategies to tailor mechanical properties, matrix composition, and degradation kinetics to guide tissue-specific repair (Figure 4C) [20]. These ECM scaffolds can be used alone or in combination with synthetic polymers and bioactive molecules to engineer hybrid constructs with improved mechanical robustness and biological performance [17,20].

Perspective and limitations. Clinically, ECM-based biomaterials are attractive because they present native biochemical motifs and can promote constructive remodeling, but translation is constrained by batch-to-batch variability, incomplete decellularization (residual DNA/lipids), donor- and tissue-source heterogeneity, sterilization-induced loss of bioactivity, limited mechanical robustness for load-bearing sites, and regulatory requirements for reproducible composition and safety. Hybrid designs that combine dECM with defined synthetic polymers and standardized manufacturing/quality-control pipelines may help address scalability and consistency while retaining ECM instructiveness [16,17,18,19,20,33].

### 5.3. ECM-Mimetic Hydrogels and Nanomaterial-Based Matrices

ECM-mimetic hydrogels aim to recapitulate key structural and biochemical features of native matrix while allowing precise control over stiffness, viscoelasticity, porosity, and ligand presentation (Figure 4B). Nicolas et al. described fundamental concepts and applications of 3D ECM mimics for mechanobiology and drug screening, emphasizing how tunable hydrogels capture disease-relevant mechanical states [33]. Kim and Cha reviewed extracellular-matrix-mimetic hydrogels based on nanomaterials, highlighting how nanoparticles, nanofibers, and supramolecular assemblies can be integrated into hydrogels to provide hierarchical architectures, dynamic crosslinking, and responsive properties [22]. These systems are particularly relevant for controlled delivery of growth factors, small molecules, and cells, as well as for engineering disease models for drug testing. Wang et al. focused on ECM-based bioinks for 3D bioprinting, summarizing how native and decellularized ECM components can be formulated into printable inks to construct anatomically relevant, cell-laden tissues with appropriate mechanical and biochemical characteristics [6,7,9,26,27,41,42].

## 6. ECM in Nano, Cell, and Gene Therapies

ECM considerations are increasingly embedded into the design of biologics, cell therapies, and nanomedicines. ECM-mimetic hydrogels and scaffolds serve as delivery platforms for stem and progenitor cells, shielding them from hostile microenvironments while providing instructive cues that promote engraftment and functional integration [17,20,33,43]. For example, ECM-mimetic hydrogels based on nanomaterials can be engineered to release chemokines and growth factors in response to local enzymatic or mechanical cues, thereby enhancing tissue-specific regeneration [22]. Nanoparticles and antibody–drug conjugates can be functionalized with ECM-binding peptides or antibodies to target fibrotic or tumor-associated ECM components, improving local drug accumulation while reducing off-target toxicity [6,8,22,29,30,31,32]. ECM remodeling enzymes and ECM-bound growth factors also influence viral vector diffusion, transduction efficiency, and off-target distribution in gene therapy. Rationally modifying ECM composition or stiffness may thus improve vector delivery and safety profiles in cardiac, hepatic, or neuromuscular gene therapies, although this area remains relatively underexplored compared with regenerative scaffolds and nanomedicines.

## 7. ECM-Derived Biomarkers, Imaging, and Integrative Diagnostics

### 7.1. Circulating ECM Fragments and Neo-Epitopes

**Figure 5:**
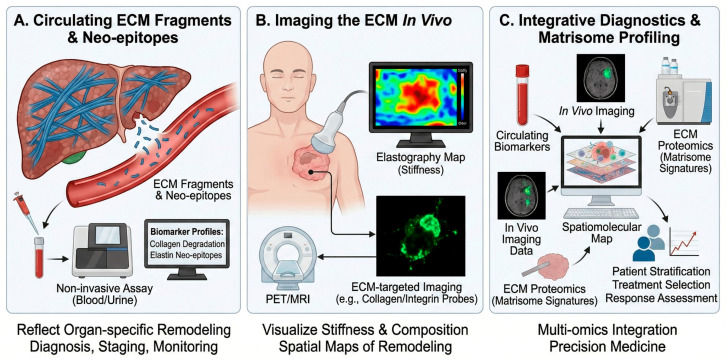
ECM-derived biomarkers, imaging, and integrative diagnostics. (**A**) Circulating extracellular matrix (ECM) fragments and neo-epitopes released during tissue remodeling can be quantified using minimally invasive blood/urine assays to generate biomarker profiles that support diagnosis, staging, and longitudinal monitoring. (**B**) In vivo ECM assessment using stiffness mapping (e.g., elastography) and molecular imaging modalities (e.g., PET/MRI) with ECM-targeted probes (e.g., collagen- or integrin-directed agents) enables spatial visualization of ECM composition and remodeling dynamics. (**C**) Integrative diagnostics combining circulating biomarkers, in vivo imaging data, and ECM proteomics (matrisome signatures) yields spatiomolecular maps that inform patient stratification, treatment selection, and response assessment in precision medicine.

ECM turnover generates fragments and neo-epitopes that can be quantified in blood, urine, or other biofluids (Figure 5A). These biomarkers reflect organ-specific matrix remodeling and can serve as non-invasive tools for diagnosis, staging, and monitoring of chronic diseases [4,22]. Karsdal et al. [21] summarized the development of ECM-associated diagnostics, including assays for collagen formation and degradation fragments, elastin and laminin neo-epitopes, and composite biomarker panels that capture fibroinflammatory activity across organs [22]. Such assays are being applied in liver, lung, kidney, cardiovascular, and musculoskeletal diseases, as well as in oncology, to stratify patients and monitor treatment response. Boaru et al. highlighted how ECM dysregulation in aging and calcification produces characteristic fragment signatures that could be exploited as mechanistically grounded biomarkers [4,8].

### 7.2. Imaging the ECM In Vivo

Advances in molecular imaging enable visualization of ECM components, crosslinking, and stiffness in vivo using MRI, PET, SPECT, and ultrasound-based elastography (Figure 5B). ECM-targeted contrast agents (e.g., collagen-specific MRI probes, integrin-targeted PET tracers) provide spatial maps of matrix remodeling in tumors and fibrotic organs, complementing circulating biomarkers [22,23]. Vedarethinam and colleagues proposed an “integrative diagnostics” paradigm in which ECM imaging is combined with mass spectrometry-based matrisome profiling and multi-omics analyses to build spatiomolecular maps of cancer ECM remodeling (Figure 5C) [6,7,8,9,23,24,25,28]. This approach supports early detection, patient stratification, and response assessment in precision oncology.

Mechanistically, ECM probes achieve specificity either by binding matrix macromolecules directly (e.g., collagen- or elastin-binding peptides/antibodies) or by targeting cell-surface receptors whose expression reflects ECM remodeling (e.g., integrins). Collagen-targeted PET probes such as the collagen-binding peptide tracer ^68^Ga-CBP8 bind exposed collagen in fibrotic lesions, enabling quantitative mapping of active collagen deposition and serving as a pharmacodynamic readout in anti-fibrotic therapy studies [49,50]. Similarly, collagen-specific molecular MRI probes (e.g., the gadolinium-based peptide probe EP-3533) accumulate in collagen-rich fibrotic tissue to report spatial heterogeneity of matrix remodeling with high anatomical resolution [51]. For the ECM–cell interface, RGD-based PET tracers (e.g., ^18^F-galacto-RGD) bind activated integrins such as αvβ3 on angiogenic endothelium and tumor cells, providing a receptor-level surrogate of remodeling and invasive potential (Table 3) [52]. Together with stiffness mapping by elastography, these probes support precise, targetable in vivo ECM phenotyping (Figure 5B) and can be integrated with circulating biomarkers and matrisome proteomics for spatiomolecular stratification [22,23].

### 7.3. ECM Proteomics and Immuno-Oncology

Day et al. reviewed ECM-focused proteomics in immuno-oncology, emphasizing that matrisome signatures in tumors correlate with immune infiltration, immunosuppressive niches, and response to checkpoint blockade [24,25,28,43]. By resolving ECM composition and post-translational modifications, proteomics can identify targetable ECM components and generate candidate biomarkers for immunotherapy response.

## 8. Conclusions and Future Directions

Viewed as a cross-disease mechanobiological axis, ECM remodeling provides measurable targets for intervention and a rationale for ECM normalization strategies that can be paired with biomaterials and diagnostic readouts to enable precision medicine. The ECM has emerged from the background of histological sections to become a central, actionable axis in human disease and therapy. Foundational and recent work converge on several key themes [1,2,53,54]:

- ECM as an active disease driver. Pathological matrix remodeling, driven by chronic inflammation, senescence, and dysregulated mechanotransduction, underpins fibrosis, calcification, and tumor progression across organs [4,5,12,13,14,29]. Targeting ECM biology is therefore essential for modifying disease trajectories, not merely managing late complications [1,2,3,5,8,10,11,12].
- Convergence of ECM biology and biomaterials science. ECM-derived scaffolds, decellularized matrices, and ECM-mimetic hydrogels now constitute a robust toolbox for tissue engineering, organ repair, and in vitro disease modeling [16,17,18,19,20,33,41]. Their modularity enables integration of cells, biologics, and nanomedicines into clinically relevant constructs tailored to specific tissues and indications [1,2,16,17,26,27].
- ECM-informed precision diagnostics. Circulating ECM fragments, ECM-targeted imaging probes, and matrisome-centered proteomics are redefining how clinicians can detect and monitor tissue remodeling [4,21,22,23,43]. Integrative frameworks that combine these modalities with genomics and transcriptomics are particularly promising for precision oncology and chronic fibrotic diseases [8,23,24,25,28].
- ECM-aware therapeutic development. Incorporating ECM metrics into early-phase clinical trials—whether as enrichment biomarkers, pharmacodynamic readouts, or surrogate endpoints—can de-risk drug development and support mechanism-based patient stratification, in line with translational aims of the field [22].
- AI-enabled ECM analytics and design. Rapid advances in computer vision and machine learning enable automated quantification of collagen architecture and ECM signatures from histology and label-free microscopy (e.g., SHG), integration of matrisome-scale proteomics with clinical phenotypes for biomarker discovery, and data-driven optimization of ECM-mimicking hydrogels and bioinks for bioprinting and regenerative applications [55,56].

Key open questions remain, including whether ECM remodeling is primarily an initiating driver or a self-reinforcing consequence of chronic pathology in different organs; resolving this will require causal, longitudinal studies integrating spatial multi-omics with quantitative mechanics and validated clinical biomarkers. Future priorities include: (i) mapping tissue- and disease-specific “mechanobiological codebooks” that link ECM mechanics and composition to cellular states; (ii) integrating ECM metrics with multi-omics at single-cell and spatial resolution; (iii) designing ECM-normalizing therapies that restore homeostatic architecture without compromising necessary repair; and (iv) standardizing ECM biomarkers and imaging protocols for regulatory-grade clinical use. By bridging basic matrix biology with biomaterial engineering, nanotechnology, and clinical research, the ECM field is well positioned to deliver the next generation of biomedicines—ranging from ECM-guided cell therapies and smart hydrogels to matrisome-informed diagnostics and ECM-targeted therapeutics. This multifaceted translational potential makes the ECM a particularly compelling focus for future work in this area [1,2,16,17,24,25,28].

## Figures and Tables

**Table 1:** ECM alterations and translational implications across major disease contexts.

Context	Key ECM Features	Translational Implications
Fibrosis/cardiovascular disease	Excess deposition, crosslinking, stiffening; persistent myofibroblast activity	Anti-fibrotic and anti-crosslinking strategies (e.g., TGF-*β*, LOX/LOXL); stiffness/turnover biomarkers [4,5,11,12,14,22]
Cancer (tumor microenvironment)	Dense/aligned collagen, heterogeneous stiffness; protease- and LOX-driven remodeling; immune exclusion	ECM normalization or controlled degradation; integrin/FAK mechanotransduction targeting; combination therapy to improve delivery/response [7,29,30,31,32,36,39]
Metabolic/adipose disorders	Fibrotic adipose ECM constraining expansion; chronic low-grade inflammation	Target ECM remodeling to improve adipose function and insulin sensitivity; monitoring via remodeling markers [40]
Regeneration/biomaterials	Need for tissue-specific biochemical cues and architecture	dECM scaffolds, ECM hydrogels/bioinks, hybrid constructs for repair and modeling [16,17,18,20,41,42]
Diagnostics/precision medicine	Disease-specific matrisome remodeling signatures	Circulating fragments + ECMtargeted imaging + proteomics for stratification/monitoring [21,23,24,25,28,43]

**Table 2:** Representative clinical-stage ECM-modulating approaches and translational rationale.

Strategy/Agent	Primary ECM/Axis	Clinical Context (Example)	Translational Rationale/Readout
Simtuzumab (anti-LOXL2)	Collagen crosslinking/stiffening	Idiopathic pulmonary fibrosis (phase 2)	Reduce pathologic crosslinking; monitor ECM turnover biomarkers
Pegvorhyaluronidase alfa (PEGPH20)	Hyaluronan-rich stroma	Metastatic pancreatic cancer (phase 3)	Decompress stroma to improve perfusion/drug delivery
Defactinib (FAK inhibitor)	Integrin–FAK mechanotransduction	Malignant pleural mesothelioma (phase 2)	Disrupt stiffness-driven signaling; combine with other therapies
Integrin-targeted imaging (RGD PET)	αvβ3 integrin (ECM–cell interface)	Solid tumors (clinical PET)	Quantify ECM–cell signaling/angiogenesis noninvasively

**Table 3:** Representative ECM-targeted imaging probes and what they report.

Probe/Modality	Target	Mechanism of Targeting	Representative Application/Readout
^68^Ga-CBP8 PET	Collagen	Collagen-binding peptide binds exposed collagen in fibrotic ECM	Quantify fibrotic burden and therapy response [49,50]
EP-3533 MRI	Collagen	Gd-chelate peptide probe accumulates in collagen-rich fibrotic tissue	Spatial mapping of liver fibrosis severity [51]
^18^F-galacto-RGD PET	αvβ3 integrin	RGD peptide binds activated integrins at the ECM–cell interface	Assess angiogenesis/invasion-associated signaling [52]

## Data Availability

No new data were created or analyzed in this study.

## Abbreviations

The following abbreviations are used in this manuscript:

ECM	Extracellular matrix
GAGs	Glycosaminoglycans
MMPs	Matrix metalloproteinases
ADAMTS	A disintegrin and metalloproteinase with thrombospondin motifs
LOX	Lysyl oxidase
LOXL	Lysyl oxidase-like
TIMPs	Tissue inhibitors of metalloproteinases
YAP	Yes-associated protein
TAZ	Transcriptional coactivator with PDZ-binding motif
SASP	Senescence-associated secretory phenotype
DAMPs	Damage-associated molecular patterns
FAK	Focal adhesion kinase
TGF-*β*	Transforming growth factor-beta
MRI	Magnetic resonance imaging
PET	Positron emission tomography
SPECT	Single-photon emission computed tomography
NK	Natural killer
PDAC	Pancreatic ductal adenocarcinoma
dECM	Decellularized extracellular matrix
